# Diagnosis of giant cell arteritis by head-contrast three-dimensional computed tomography angiography: two case reports

**DOI:** 10.1186/s13256-019-2199-0

**Published:** 2019-09-11

**Authors:** Toshio Kawamoto, Michihiro Ogasawara, Souichiro Nakano, Yuko Matsuki−Muramoto, Masakazu Matsushita, Kenjiro Yamanaka, Ken Yamaji, Naoto Tamura

**Affiliations:** 1 Department of Internal Medicine and Rheumatology, Juntendo University, School of Medicine, 2-1-1 Hongo, Bunkyo-ku, Tokyo, 113-8421 Japan; 2 Juntendo Tokyo Koto Geriatric Medical Center, 3-3-20 Shinsuna, Koto-ku, Tokyo, 136-0075 Japan; 3 Juntendo University Koshigaya Hospital, 560 Fukuroyama, Kosigaya-shi, Saitama, 343-0032 Japan

**Keywords:** Giant cell arteritis, 3D computed tomography angiography, A temporal artery biopsy, Case report

## Abstract

**Introduction:**

Temporal artery biopsy is essential for the diagnosis of giant cell arteritis. It has been shown that ^18^F-fluorodeoxyglucose positron emission tomography-computed tomography, magnetic resonance angiography, and ultrasonography are useful for the diagnosis of giant cell arteritis. However, there are only a few reports on the usefulness of three-dimensional computed tomography angiography in the diagnosis of giant cell arteritis. We describe two cases in which giant cell arteritis was difficult to diagnose using positron emission tomography-computed tomography and magnetic resonance angiography but was diagnosed using three-dimensional computed tomography angiography, thus showing the importance of three-dimensional computed tomography angiography in the diagnosis of giant cell arteritis.

**Case presentation:**

Case 1: An 81-year-old Japanese man. Laboratory investigations revealed normocytic anemia and raised inflammatory marker levels. Slight bleeding in the right posterior pole of his eyeball and leukoma of his left cornea were observed on fundus examination. Stenosis and stoppage of the temporal artery were detected on three-dimensional computed tomography angiography. A diagnosis of giant cell arteritis was made, and he was started on orally administered prednisolone. His headache and C-reactive protein levels improved. Four weeks after glucocorticoid steroid treatment, three-dimensional computed tomography angiography revealed improvement in stenosis and stoppage of temporal artery.

Case 2: A 74-year-old Japanese woman. A dose of 20 mg of prednisolone was administered and her polymyalgia and polyarthritis improved; however, her headache and ear occlusion persisted. Although vasculitis was not detected on positron emission tomography-computed tomography, stenosis and stoppage of the temporal artery were detected on computed tomography angiography. She was diagnosed as having giant cell arteritis and started on orally administered prednisolone treatment (60 mg daily). Her headache and C-reactive protein levels improved. Four weeks after glucocorticoid treatment, three-dimensional computed tomography angiography showed improvement in stenosis and stoppage of temporal artery.

**Conclusions:**

In both patients with giant cell arteritis, three-dimensional computed tomography angiography revealed improvement in stenosis and stoppage of temporal artery after glucocorticoid treatment. We conclude that computed tomography angiography along with magnetic resonance angiography, positron emission tomography-computed tomography, and ultrasonography are important for the diagnosis of giant cell arteritis.

## Background

It has been reported that the frequency of giant cell arteritis (GCA) in patients with polymyalgia rheumatica (PMR) is 5–30% [1]. Diagnosis of GCA is very important to determine appropriate immunosuppressive therapies for the affected patients. Temporal artery (TA) biopsy is essential for the diagnosis of GCA. In the past, it was essential to perform routine biopsies of the TA to diagnose GCA [2, 3]. However, routine biopsies are disadvantageous in that they occasionally yield false-negative results and the procedure is invasive and can be performed only in a few institutions. It has been reported that magnetic resonance angiography (MRA) is a useful method for evaluating vasculitis [4, 5]. ^18^F-fluorodeoxyglucose positron emission tomography-computed tomography (PET-CT) has rapidly become widely used these days and has been reported to be useful for diagnosing not only cancer but also vasculitis [6]. Further, reports on the usefulness of ultrasonography have also been increasing [7]. However, until now, there have only been a few reports on the usefulness of three-dimensional computed tomography angiography (CTA) in the diagnosis of GCA [8]. We aim to show the importance of three-dimensional CTA in the diagnosis of GCA.

## Case presentation

### Case 1

**Fig. 1:**
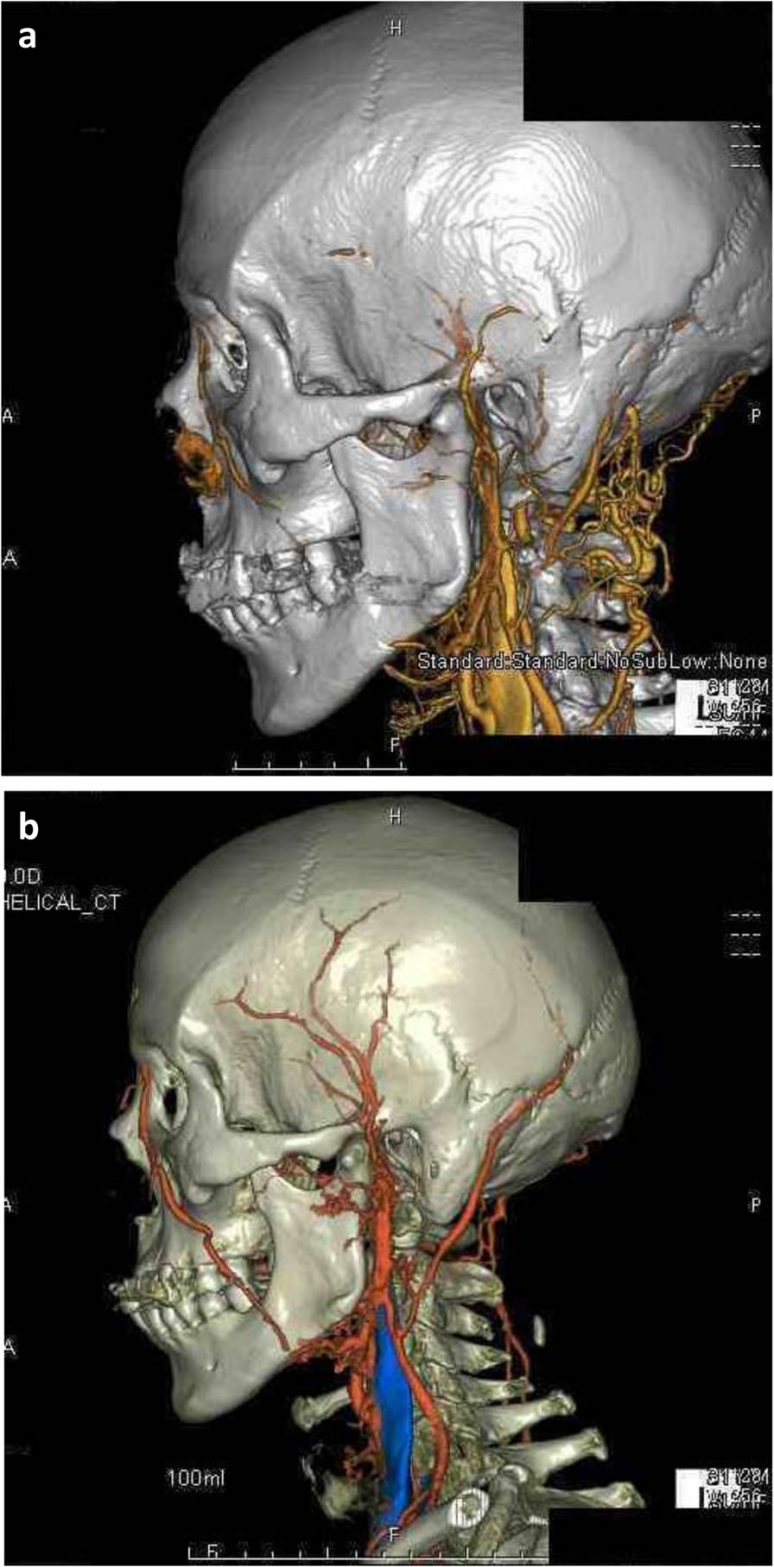
**a** Three-dimensional computed tomography angiography image of Case 1 before immunosuppressive therapy. **b** Three-dimensional computed tomography angiography image of Case 1 after therapy. Image (**a**) shows stoppage of the temporal artery, whereas (**b**) shows opening of the temporal artery

An 81-year-old Japanese man presented with a history of pharyngeal pain, malaise in both lower extremities, and jaw claudication for 1 month. He had a past medical history of hypertension, angina pectoris, and reflux esophagitis. He reported no visual disturbance. On physical examination, he was febrile with a body temperature of 38 °C. He had no active synovitis or rash. His TAs were not palpable but were tender; his cranial nerve was normal. There was no lymphadenopathy or hepatosplenomegaly. Other findings on physical examination were unremarkable. A laboratory investigation revealed normocytic anemia and raised inflammatory marker levels. His renal function and electrolyte levels were within normal reference ranges. His blood and urine cultures showed negative results. Further investigations included screening for vasculitis, autoimmune disorders, viral infections, and malignancies; the results were either negative or in the normal range (Table 1). Slight bleeding of the right posterior pole of his eyeball and leukoma of his left cornea were observed on fundus examination. Computed tomography (CT) of his neck, thorax, abdomen, and pelvis were negative for lymphadenopathy, mass, abscess, and infective foci. A left mesencephalic artery stenosis lesion was detected on MRA of his head; angiitis of his breast and abdomen was detected on PET-CT; stenosis and stoppage of the TA were detected on three-dimensional CTA (Fig. 1a). A diagnosis of GCA was made and he was started on orally administered prednisolone (PSL; 40 mg daily). His headache and C-reactive protein (CRP) levels improved. On follow-up, he showed good recovery, and his PSL dose was gradually tapered to 5 mg daily. Four weeks after glucocorticoid steroid treatment, three-dimensional CTA showed improvement of stenosis and stoppage of TA (Fig. 1b).

**Table 1:** Results of laboratory, serological, and immunological investigations

Variables	Results	Normal reference range
Hemoglobin	8.9 g/dL	14–18 g/dL
Red blood cells	2.97 10^12^/L	4.25–5.80 10^12^/L
Mean cell volume	89.7 fl	83–100 fl
Platelet count	31.3 10^9^/L	14–35 10^9^/L
White cell count	6.21 10^9^/L	3.4–9 10^9^/L
Neutrophils	4.33 10^9^/L	1.6–6.4 10^9^/L
Gamma-glutamyl transpeptidase	91 U/L	10–72 U/L
Alkaline phosphatase	359 U/L	104–295 U/L
Alanine aminotransferase	93 U/L	41–112 U/L
Lactate dehydrogenase	134 U/L	116–230 U/L
Sodium	140 mmol/L	136–145 mmol/L
Potassium	4.4 mmol/L	3.4–5 mmol/L
Urea	5.1 mmol/L	0–6.9 mmol/L
Creatinine	0.78 mg/dl	0.4–1.1 mg/dl
Erythrocyte sedimentation rate (ESR)	134 mm/hour	0–10 mm/hour
C-reactive protein (CRP)	14.6 mg/dL	0–0.3 mg/dL
Antinuclear antibody	Negative	
Rheumatoid factor	1 IU/ml	0–20 IU/ml
Anti-cyclic citrullinated peptide antibody	0.6 U/ml	0–4.4 U/ml

### Case 2

**Fig. 2:**
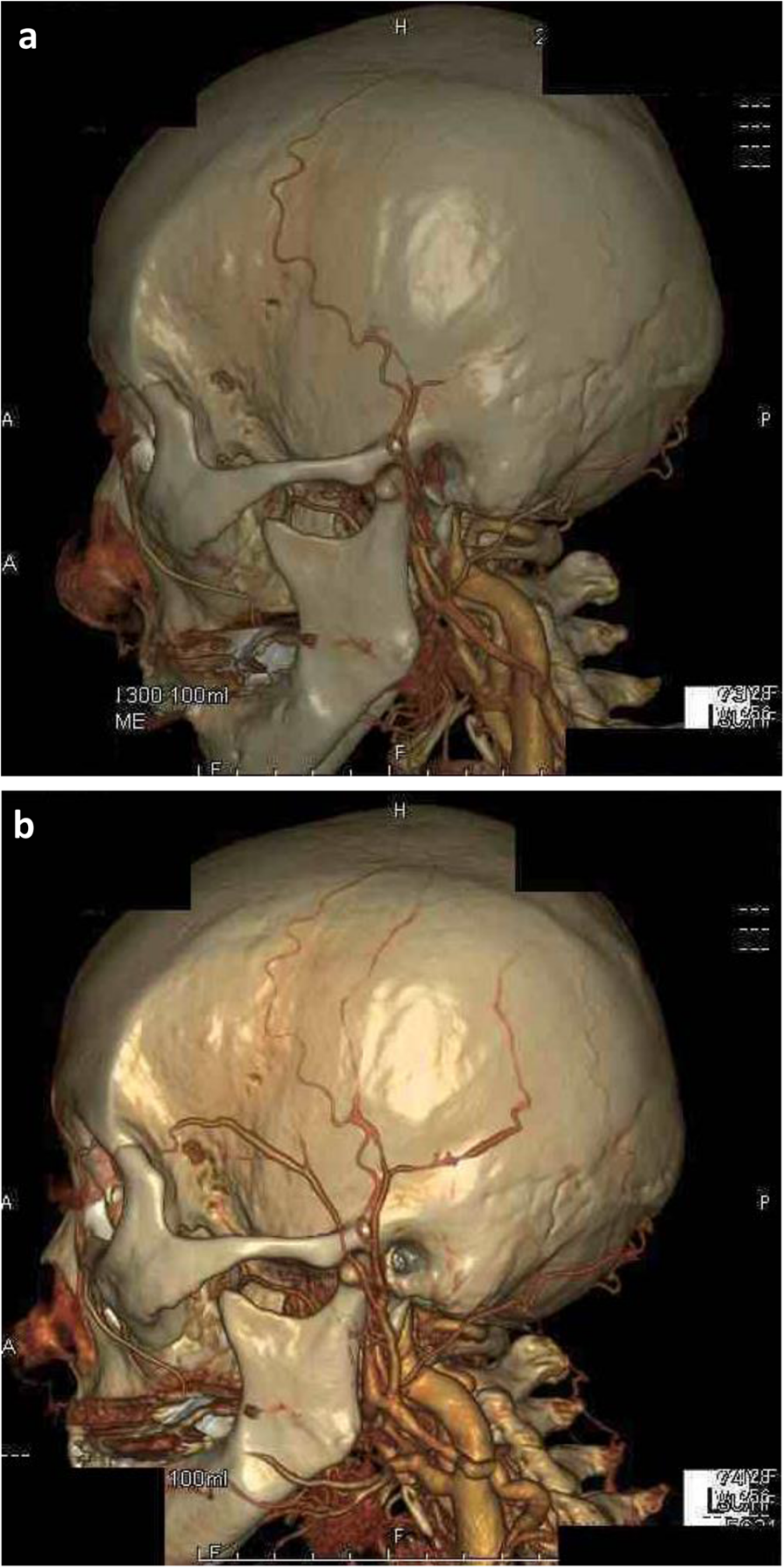
**a** Three-dimensional computed tomography angiography image of Case 2 before immunosuppressive therapy. **b** Three-dimensional computed tomography angiography image of Case 2 after therapy. Image (**a**) shows stoppage of the temporal artery, whereas (**b**) shows opening of the temporal artery

A 74-year-old Japanese woman complained of polymyalgia and polyarthritis; her pain had increased, causing headache and ear occlusion. She had a past medical history of hypertension. She reported no visual disturbance. On physical examination, she was febrile with a body temperature of 37.5 °C. Her TAs were not palpable; her cranial nerve was normal. There was no lymphadenopathy or hepatosplenomegaly. Other findings on physical examination were normal. Laboratory investigations showed normocytic anemia and raised inflammatory marker levels. Her renal function and electrolyte levels were within normal reference ranges. Her blood and urine cultures showed negative results. Further investigations included screening for vasculitis, autoimmune disorders, viral infections, and malignancies; the results were either negative or in the normal range (Table 2). Right optomeninx degeneration was observed on fundus examination. The results of a CT scan of her neck, thorax, abdomen, and pelvis were negative for lymphadenopathy, mass, abscess, and infective foci. A dose of 20 mg of PSL was administered, and her polymyalgia and polyarthritis improved; however, her headache and ear occlusion persisted. Although vasculitis was not detected on PET-CT, stenosis and stoppage of the TA were detected on three-dimensional CTA (Fig. 2a). She was diagnosed as having GCA and was started on orally administered PSL (60 mg daily). Her headache and CRP levels improved. On follow-up, she showed good recovery, and her PSL dose was gradually tapered to 5 mg daily. Four weeks after the glucocorticoid steroid treatment, three-dimensional CTA showed improvement of stenosis and stoppage of TA (Fig. 2b).

**Table 2:** Results of laboratory, serological, and immunological investigations

Variables	Results	Normal reference range
Hemoglobin	12.0 g/dL	14–18 g/dL
Red blood cells	387	425–580
Mean cell volume	92.6	83–100
Platelet count	90.5	14–35
White cell count	16,800	3400–9000
Neutrophils	13,300	1600–6400
Gamma-glutamyl transpeptidase	45	10–72 U/L
Alkaline phosphatase		104–295 U/L
Alanine aminotransferase	34	41–112 U/L
Lactate dehydrogenase	263	116–230 U/L
Sodium	140	136–145 mmol/L
Potassium	4.3	3.4–5 mmol/L
Urea	5.2	0–6.9 mmol/L
Creatinine	0.54	0.4–1.1
Erythrocyte sedimentation rate (ESR)	79	0–10 mm/hour
C-reactive protein (CRP)	10.7	0–0.3 mg/dL
Antinuclear antibody	Negative	
Rheumatoid factor	63 IU/ml	0–20 IU/ml
Anti-cyclic citrullinated peptide antibody	2.6 U/ml	0–4.4 U/ml

## Discussion

We believe that three-dimensional CTA is a useful method to diagnose GCA because of the following reasons:

First, both cases presented with stenosis and stoppage of blood vessels. It has been reported that three-dimensional CTA is a useful method for estimation of lumen and for discrimination of vasculitis and arteriosclerosis [8]. Moreover, three-dimensional CTA provides the advantage of visually confirming the findings by constructing three-dimensional images.

Second, an improvement in the three-dimensional CTA findings was observed after steroid administration in both cases (Figs. 1b and 2b). This means that three-dimensional CTA is highly sensitive to change and is useful for estimation of the effectiveness of treatment.

Third, although MRA and PET-CT yielded negative GCA findings in Case 1, three-dimensional CTA yielded positive findings for GCA. In Case 2, PET-CT yielded negative GCA findings, whereas three-dimensional CTA yielded positive findings for GCA.

Although there have been studies comparing the efficacies of MRA, PET-CT, and ultrasonography, only a few studies have compared the efficacy of three-dimensional CTA with that of the other methods [8]. These studies showed that three-dimensional CTA is a useful method for the diagnosis of GCA along with PET-CT and MRA. However, examination using three-dimensional CTA has disadvantages such as the need for contrast medium, and a previous study has reported that three-dimensional CTA is not applicable for early diagnosis [8].

Although TA biopsies are useful for establishing a diagnosis of GCA, it is difficult to examine all cases using this technique because this technique can only be performed in a few institutions and because it takes a long time to obtain the results [1–3]. Although three-dimensional CTA is superior in estimating the lumen and blood vessel thickening and blood vessel lesions in both intracranial and extracranial vessels, it requires a contrast medium and has a low sensitivity for early blood vessel lesions [1–3, 8]. Although MRA is superior in estimating blood vessel thickening and edematous lesions, it cannot detect cranial blood vessel lesions on the surface [1–5]. Although PET-CT is superior in early estimation of blood vessel lesions, it is difficult to perform, does not clearly discriminate arteriosclerosis, and cannot detect blood vessel lesions in a cranial vessel [1–3, 6]. Although ultrasonography can be performed easily and can estimate blood vessel lesions in both intracranial and extracranial vessels, it depends on the ability of the equipment and the evaluator and cannot clearly discriminate arteriosclerosis [1–3, 7].

## Conclusions

We believe that three-dimensional CTA along with MRA, PET-CT, and ultrasonography are useful in the diagnosis of GCA. There are advantages and disadvantages to each examination method. We aim to diagnose GCA using ultrasonography initially, followed by three-dimensional CTA and MRA. TA biopsies may be performed on the basis of the results from other examinations [9]. In the future, it will be preferable to examine how each imaging modality contributes to the diagnosis of GCA and to clarify the characteristics of each imaging modality when the imaging modalities are combined.

## Data Availability

All data generated or analyzed during this study are included in this published article.

## Abbreviations

CRP: C-reactive protein
CT: Computed tomography
CTA: Computed tomography angiography
GCA: Giant cell arteritis
MRA: Magnetic resonance angiography
PET-CT: Positron emission tomography-computed tomography
PMR: Polymyalgia rheumatica
PSL: Prednisolone
TA: Temporal artery
